# Mechanics and spiral formation in the rat cornea

**DOI:** 10.1007/s10237-014-0592-6

**Published:** 2014-06-05

**Authors:** T. Mohammad Nejad, S. Iannaccone, W. Rutherford, P. M. Iannaccone, C. D. Foster

**Affiliations:** 1 Department of Civil and Materials Engineering, MC 246, 3085 Engineering Research Facility, University of Illinois at Chicago, 842 W. Taylor St., Chicago, IL 60607 USA; 2 Children’s Memorial Research Center, Northwestern University, 2430 N Halsted St., Chicago, IL 60614 USA; 3 Department of Mathematics, Evanston Township High School, 1600 Dodge Ave., Evanston, IL 60201 USA

**Keywords:** Spiral, Rat cornea, Chimera, Cornea mechanics, Finite element analysis

## Abstract

During the maturation of some mammals such as mice and rats, corneal epithelial cells tend to develop into patterns such as spirals over time. A better understanding of these patterns can help to understand how the organ develops and may give insight into some of the diseases affecting corneal development. In this paper, a framework for explaining the development of the epithelial cells forming spiral patterns due to the effect of tensile and shear strains is proposed. Using chimeric animals, made by combining embryonic cells from genetically distinguishable strains, we can observe the development of patterns in the cornea. Aggregates of cell progeny from one strain or the other called patches form as organs and tissue develop. The boundaries of these patches are fitted with logarithmic spirals on confocal images of adult rat corneas. To compare with observed patterns, we develop a three-dimensional large strain finite element model for the rat cornea under intraocular pressure to examine the strain distribution on the cornea surface. The model includes the effects of oriented and dispersed fibrils families throughout the cornea and a nearly incompressible matrix. Tracing the directions of critical strain vectors on the cornea surface leads to spiral-like curves that are compared to the observed logarithmic spirals. Good agreement between the observed and numerical curves supports the proposed assumption that shear and tensile strains facilitate sliding of epithelial cells to develop spiral patterns.

## Introduction

The cornea is the transparent, anterior layer of the eye that protects the eye and refracts light. It is covered with a tear film that, together with the underlying tissue, provides almost two-thirds of the refractive power of the eye (DelMonte and Kim 2011). The curvature and material composition of cornea are largely responsible for its refractive power, and a slight change in its structure remarkably affects the vision function.

In many mammals, including humans, mice, and rats, the cornea consists of five layers. From the anterior to the posterior, the layers are the epithelium, Bowman’s membrane, the stroma, Descemet’s membrane, and the endothelium. The epithelium is the outermost layer of the cornea and consists of cells that can reproduce and regenerate after an injury. The endothelium consists of non-renewable cells that keep cornea hydrated and transparent. Permeability of the endothelium can affect the transparency of the cornea and its ability to function correctly.

The stroma is the thickest layer of the cornea and contributes the majority of the mechanical strength and stiffness of the cornea. It is composed of a complex network of collagen fibrils that are distributed in a nearly incompressible and viscoelastic matrix. The collagen fibrils bundle together to form fibers that are stacked parallel to create layers called lamellae. The fibers act as reinforcement to the cornea and provide mechanical strength along their orientation. The collagen fibrils are distributed in all possible orientations in the plane of the cornea but have preferred orientations around the center and the edge (Meek et al. 1987; Aghamohammadzadeh et al. 2004). For example, in the human cornea, collagen is preferentially distributed along the inferior–superior and nasal–temporal directions around the center and circumferential and radial around the limbus (a region at the corneal periphery where it meets the sclera). Fibrils exhibit horizontal preferred orientation at the center and circumferential around the edge in the mouse cornea (Hayes et al. 2007; Sheppard et al. 2010). The arrangements of collagen fibrils play important role in the mechanical strength of cornea. The dominant orientations of the collagen fibrils throughout the lamellae lead to anisotropy of the cornea material.

The cornea has a complex structure with non-uniform thickness throughout. The outer and inner surfaces of the cornea have different curvatures. The central thickness of the human cornea is thinner than the thickness around the limbus. However, in some animals such as mice and rats, cornea is thicker at the center. The curvature and thickness of cornea are largely responsible for the refractive power, and a slight change in corneal structure remarkably affects vision. Hence, studying the mechanical behavior of the cornea is critical in investigating vision and possible disorders.

In vivo and in vitro studies have been performed on the cornea to investigate the biomechanical behavior of the tissue. Recently, numerical techniques such as finite element method (FEM) have been widely used as an effective and noninvasive technique to examine the cornea mechanics. FEM has been implemented to model deformation of the cornea for surgery simulation and refractive surgery planning (Pandolfi et al. 2009; Roy and Dupps 2009). Some authors (Gefen et al. 2009; Carvalho et al. 2009; Foster et al. 2013) used FEM to investigate biomechanical interactions in corneal diseases such as keratoconus in order to better understand the etiology of the illness and offer possible treatment solutions. Discussed in Jo and Aksan (2010), Shafahi and Vafai (2011), FEM is implemented to study response of the cornea to thermal treatments and disturbances. Some others (Mandel et al. 2012; Guimera et al. 2010) used FEM to predict the electrical properties of the corneal endothelium and study variations in the permeability. Various applications of FEM in studying corneal biomechanical behavior, surgery predictions, clinical application, investigating related diseases, and the response of the cornea to impact have been reviewed (Mohammad Nejad et al. in press).

During the generation of an organ or tissue, stem cells are allocated to areas that are responsible for production of the tissue or organ. The cells then distribute themselves in the developing organ or tissue as a result of cell division, cell movement, and cell death (Iannaccone 1987). The resulting distribution of cells into an organ or tissue can be observed when two or more genetically distinct populations of cells that can be visually distinguished comprise the tissues of an animal. Aggregation chimeras (Prather et al. 1989) provide a system that allows such visualization. Chimeras in the biological sense are experimentally produced combinations of biological materials that do not naturally occur together. Chimeric animals are animals made by combining early embryos derived from at least two different strains. Therefore, at least four parents contribute, that is there are at least two pregnancies that give rise to two distinguishable embryos. Because of this, the animals are also known as tetraparental animals. These animals are produced by allowing the individual cells of early embryos from two genetically distinct strains to mix prior to implantation. This is done by physically amalgamating the two distinct embryos into one large embryo, which is allowed to develop to term. In very early stages of development, there is a compensation for the total number of cells present that results in a normal animal at term. The resulting animal has two genetically distinct lineages (one from each set of parents), and these lineages can be observed microscopically utilizing a variety of markers, in this case enhanced green fluorescent protein (eGFP) was used to mark one of the lineages. Aggregates of cell progeny from one lineage or the other, called patches, form as organs and tissues develop. The patches form distinct patterns that are different in different tissues, but in a given tissue, are the same in different individual chimeras and even in different species. That is, the pattern observed in mouse chimeras is also seen in rat chimeras in a given tissue. The patterns arise from cell division, cell movement, and cell death (Landini and Iannacconne 2000).

In the cornea, the allocation of epithelial cells is followed by an assortment of cells such that a distinctive pinwheel pattern is observed in both mouse and rat (Collinson 2004; Iannaccone et al. 2012). Moreover, the edges of patches of cells of similar lineages trace out characteristic spiral curves that have been established as logarithmic spirals (Iannaccone et al. 2012). The patterns of cell assortment correlate with the distribution of nerves in the cornea (Dvorscak and Marfurt 2008) implying that there is some global process or force that is responsible for the distribution.

In the same way as for other organ mosaic patterns, it was held that stem cell divisions laying down a trail of progeny as the cornea expanded was responsible for the spiral assortment of epithelial cells. However, a number of fine details of the timing and distribution of cells make that explanation unlikely (Iannaccone et al. 2012). Since the absence of spiral distribution correlates with human disease states and abnormal cornea structure and function, the possibility that biomaterial properties contribute to normal epithelial cell distribution is of significance.

Studying the development of epithelial cells into spiral patterns is important as it can reveal greater understanding of corneal function and possible disorders. While some models have been proposed to explain formation of spiral patterns, none have been able to completely explain this phenomenon. This paper proposes a framework for explaining assortment of epithelial cells into spiral patterns due to the effect of stresses and strains on the cornea.

To this end, a large deformation model for the rat cornea that includes the stiffening effect of oriented and dispersed collagen fibrils in incompressible matrix is developed and implemented in a finite element code to investigate the stresses and strains on the surface of the cornea subjected to intraocular pressure. The directions of critical strain, in particular, tend to form spiral-like curves that are compared with logarithmic spirals fitted to the epithelial cell boundaries on the rat cornea confocal images.

The remainder of this paper is organized as follows: Sect. 2 discusses the special assortment of epithelial cell into spiral patterns on the surface of the rat cornea along with observations and measurements of those patterns. In Sect. 3, an anisotropic and large strain finite element model of the rat cornea is developed. This includes creating the three-dimensional geometric model of the rat cornea, and an overview of the restrictions applied on the cornea limbus. The section is followed by a review of the preferred orientation of collagen fibrils in some mammalian cornea as well as assumption of predominant fibril directions on the rat cornea. Also, a structural constitutive formulation including the stiffening effects of collagens in Neo-Hookean matrix is discussed. Section 4 focuses on the spiral post-processing, which includes the determination of in-plane strains and developing an algorithm for tracking directions of critical strains. In Sect. 5, the numerical simulation results are compared to experimental observations. This paper is followed by a discussion on potential mechanical explanation for spiral formation in Sect. 6, and finally, conclusion is given in Sect. 7.

## Spirals in the rat cornea

Rat chimeras were produced as described previously by amalgamation of two 8-cell morulae, one of which ubiquitously expresses eGFP. Chimeras were perfused with 4 % paraformaldehyde in phosphate-buffered saline (PBS), a physiological saline solution, and eyes were enucleated and kept in 4 % paraformaldehyde at $4\,^{\circ }\hbox {C}$ overnight. Corneas were dissected then relaxed with small radial incisions and imaged on a Zeiss 510 META confocal microscope. Since confocal microscopy uses a variable pinhole in front of the photomultiplier tube, light outside of the focal plane of the objective is blocked from detection. Varying the diameter of the confocal pinhole allows for a change in the thickness of the focal plane. Imaging relaxed corneas, which now lie flat and parallel with the plane of focus, allowed for easy optical separation of epithelium fluorescence without interference or contribution from cells in the stroma or endothelium by simply adjusting the focal plane and its thickness to match that of the epithelium. Because the scale of the corneas is much larger than the field of view of the microscope’s objective, multiple images were taken using a computer-controlled stage and automatically stitched together in software.

Landmarks on the images were picked along cell lineage boundaries, and coordinates of edge landmarks were recorded. Using an initial guess of the spiral center, angle $(\theta )$ and radii ($r$) were calculated for each landmark from this initial center. The polar coordinate data of landmarks were then fit using exponential curves of the form $r = ae^{b\theta }$, which are the polar equations of logarithmic spirals.

**Fig. 1:**
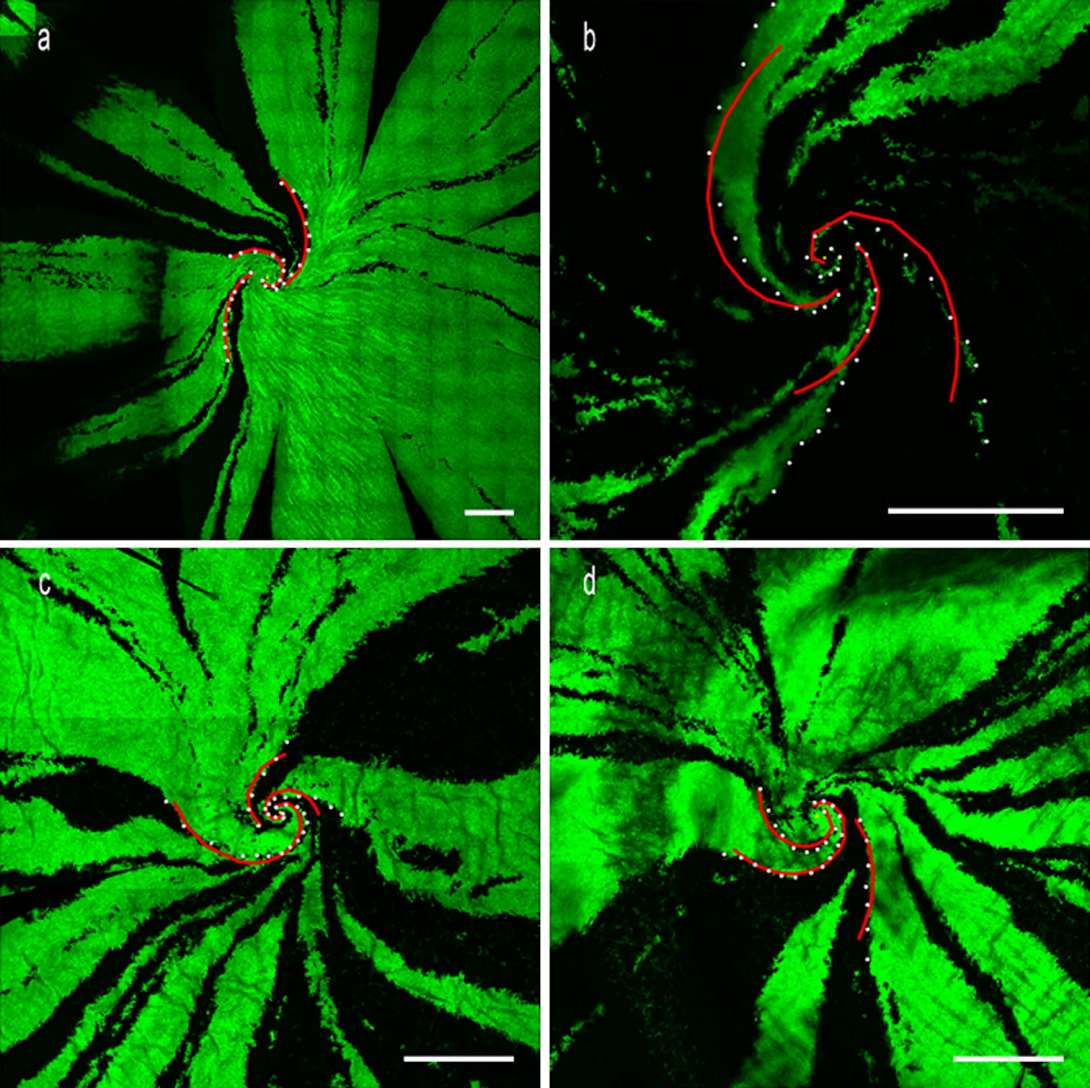
Confocal images of cornea from **a** 10-month-old rat (C1), **b** 13-month-old rat (C2), **c**, **d** 16-month-old rat (C3-1 and C3-2, right and left corneas of the same animal, respectively). For each rat cornea, three sets of landmarks are overlaid along the cell boundaries that resemble spiral patterns. These points are fitted with logarithmic spirals (*red curves*), and the pitch angles are measured. *Scale bars* on each cornea are $500\,\upmu \hbox {m}$

The exponential equation used to fit the landmark data was used to calculate radii of the fit at the original angles of the landmarks. Total error between the fit and the original landmarks was used to optimize the spiral’s center (Table 1). The radii and angles were then recalculated and refit with exponential curves using this optimized center. Four cornea images from three rats of ages 10, 13, and 16 months old were analyzed. Three edges per image were fitted with logarithmic spirals and are demonstrated in Fig. 1.

**Fig. 2:**
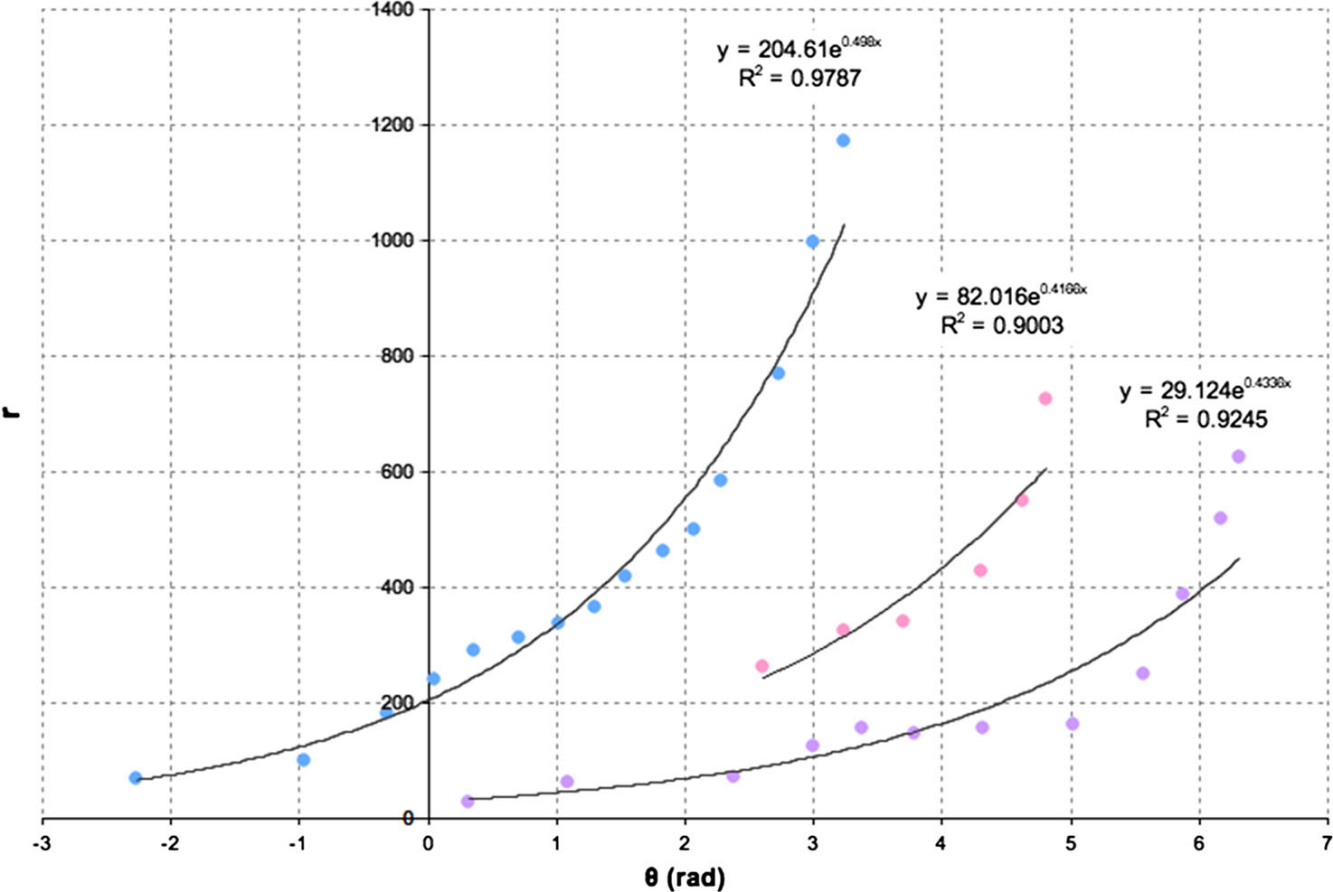
$r$ versus $\theta$ plot of landmark points and the matching logarithmic spirals in the polar coordinates for sampling cornea C3-1

**Fig. 3:**
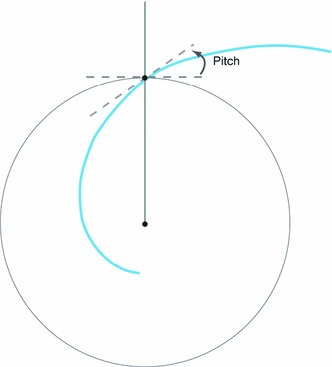
Schematic representation of pitch angle of a logarithmic spiral

Figure 2 depicts the landmark points and the matching logarithmic spirals in the polar coordinates for cornea C3-1 ($r$ vs. $\theta$ plot). The variable $b$ of the exponential function fit to the landmarks is related to the curvature of the spiral and therefore is more useful as a unique descriptor of the patch edge. A smaller value of $b$ describes a spiral with a tighter curve with less increase in radius with each increment of angle around the spiral. Likewise, a larger value in $b$ describes a spiral with a looser curve where each increment in angle means a larger change in the radius. A related, but simpler description of each spiral fit is the pitch angle, which represents the angle of inclination between the tangent of the spiral and that of a circle with the same center (see Fig. 3). Hence, a pitch angle varying from zero to $\frac{\pi }{2}$ would be a curve that relaxes from a circle of radius $a$ at a pitch angle of zero to a straight radial stripe at a pitch angle of $\frac{\pi }{2}$. Spiral pitch angles reported for each fit are calculated as $\frac{\pi }{2}-\hbox {Atan}(\frac{1}{b})$. A negative pitch angle describes a spiral which moves clockwise toward the center, while spirals that move counterclockwise inward have a positive pitch angle.

**Table 1:** Results of the measurements of the logarithmic spirals fitted to cell lineage boundaries for the four sampling rat corneas

Cornea label	C1			C2			C3-1			C3-2		
Spiral No.	1	2	3	1	2	3	1	2	3	1	2	3
$b$	-0.369	-0.921	-1.225	0.555	0.688	0.636	0.498	0.434	0.417	0.446	1.049	0.413
Pitch angle ($^{\circ }$)	-20.3	-42.7	-50.8	29.0	34.5	32.5	26.5	23.4	22.6	24.0	46.4	22.4

## Rat cornea finite element model

### Geometric model of the rat cornea

**Fig. 4:**
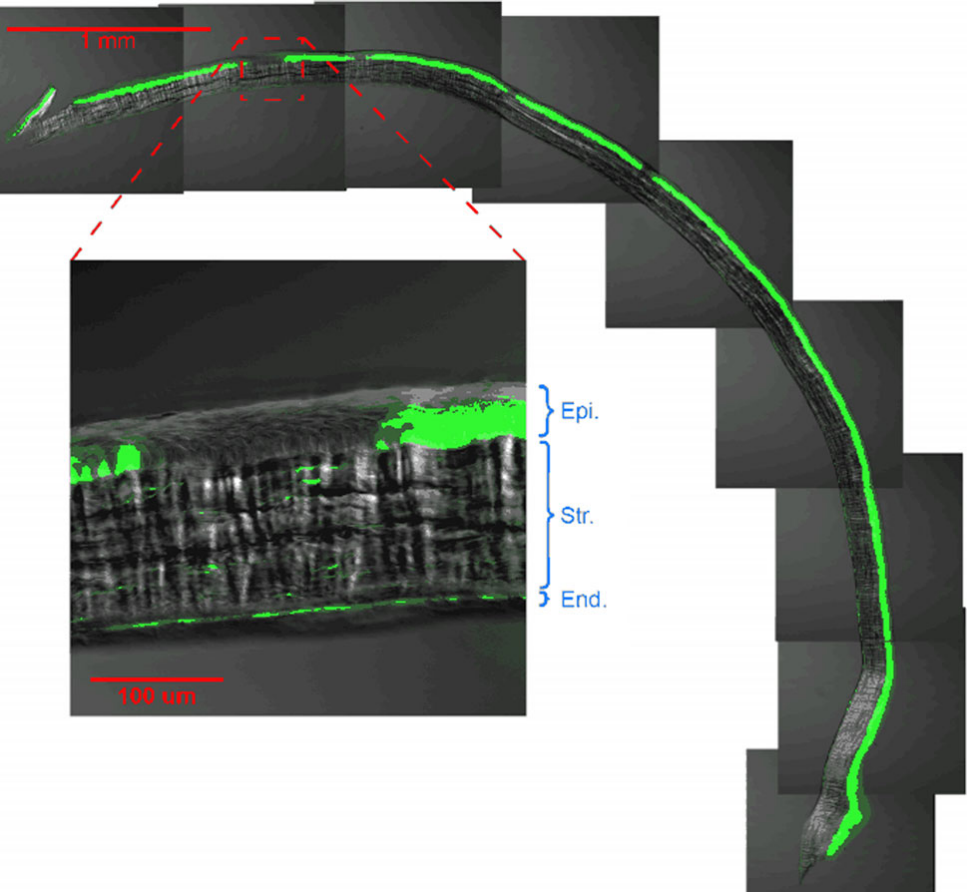
Confocal image of cross section of a rat cornea used to construct the geometry of the cornea for the finite element model (Iannaccone et al. 2012). In the *zoomed-in* image, *Epi.* is the epithelium, *Str.* is the stroma, and *End.* is the endothelium

**Fig. 5:**
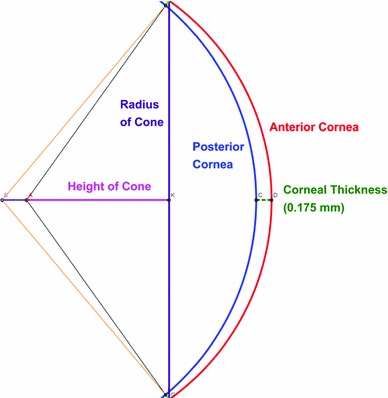
Two-dimensional representation of geometry used to create the three-dimensional structure of the rat cornea

The first step in the numerical modeling effort is development of a dimensionally correct cornea model. A rat cornea was cryosectioned, and the sections were mounted on slides before confocal imaging. Figure 4 illustrates the provided confocal image of the cross section of a rat cornea. The figure was analyzed to obtain precise measurements (given in Table 2). As seen in the table, the interior and exterior surfaces of the image were fit to spheres of different radii and centers. This leads to non-uniform thickness throughout the cornea being greatest at the apex (nearly 175 $\upmu$m). The three-dimensional structure of the rat cornea was created based on the obtained measurements (see Fig. 5).

### Boundary conditions

There is a viscous fluid behind the cornea, the aqueous humor, that exerts pressure, called intra-ocular pressure (IOP), on the cornea. Under physiological conditions, the IOP inflates the cornea and gives it shape. Some authors (Uchio et al. 1999; Amini and Barocas 2009) have modeled the entire eyeball to consider more realistic displacement at the cornea edge. However, the proposed models may be time-consuming and not economical. In addition, modeling the entire eye would require a detailed model of the limbus and sclera of the rat. In particular, the material data for those parts of the eye do not appear to be available in the literature. Some others offered an approximate boundary condition, with roller support at the edge inclined at $40^{\circ }$ with respect to the horizontal axis, to represent the cornea limbus behavior in human eye (Carvalho et al. 2009; Elsheikh et al. 2010; Fraldi et al. 2011). In this work, the displacement boundary is set up to restrict displacement at the limbus, but allow for rotation between the cornea and the limbus [similar to the model proposed by Pandolfi and Manganiello (2006)]. Since the rat cornea is thinner at the limbus than at the center, the approximation used is appropriate.

**Table 2:** Measurements obtained for creating three-dimensional rat cornea structure

Description	Calculation (mm)
Anterior radius of curvature	2.767
Posterior radius of curvature	2.869
Thickness (at apex)	0.175
Height of cone for model	1.610
Radius of cone for model	2.250

A constant intraocular pressure of 1.7 kPa (Cabrera et al. 1999) was uniformly distributed to the inner surface of the cornea. In order to maintain the correct direction (normal to the cornea surface) and magnitude of the pressure with respect to the changing surface, a follower forces technique was applied (Belytschko et al. 2000).

### Assumptions on fibril orientations

**Fig. 6:**
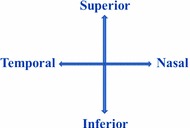
Schematic of superior–inferior and nasal–temporal directions (Meek et al. 1987)

The density and orientation of collagen fibrils in the stroma greatly affect the biomechanical behavior of the cornea, and hence studying arrangement of collagens is important to developing an accurate model (Hayes et al. 2007). Recently, X-ray scattering has been successfully used to investigate the predominant orientations of the collagen fibrils in the cornea (Meek et al. 1987; Aghamohammadzadeh et al. 2004; Hayes et al. 2007). In many mammals, collagen fibrils are oriented circumferentially (parallel to the edge of the cornea) in the region around the edge of the cornea. The dominant orientation of collagen is weaker in the central zone of the cornea and differs among mammalian species. For example, collagen fibrils are predominantly oriented along the horizontal and vertical directions (superior–inferior and nasal–temporal, Fig. 6) in the human cornea. X-ray patterns revealed vertical predominant directions for horse cornea while showing circumferential preferred orientations in pig and rabbit (Hayes et al. 2007) and horizontal directions in the mouse cornea (Sheppard et al. 2010).

Second harmonic generation (SHG) is a useful tool for studying collagen fibril orientations throughout the tissue thickness. The advantage of this approach is that orientation of collagen fibrils can be seen as a function of depth. Latour and coworkers performed SHG microscopy profiles of the rat cornea and provided depth images of collagen directions throughout the cornea thickness (Latour et al. 2012). Using the depth profiles of the central cornea, average of the predominant orientations of collagen fibrils in the entire thickness of the rat cornea was calculated. This analysis revealed four preferred directions of collagen fibrils around the center of the rat cornea: nasal–temporal, superior–inferior, nasal–superior to temporal–inferior, and superior–temporal to nasal–inferior.

While the images obtained only cover a small (about 350 microns) portion of the cornea, these directions are assumed to be predominant throughout the central region of the cornea.

To summarize the discussion on modeling of collagen fibrils in the rat cornea, for numerical purposes, the predominant directions are assumed by introducing three zones in the cornea as follows:

1. Central zone ($${r}<{R}_{\mathrm{in}}$$):
  In the central cornea, it is assumed that collagen fibrils demonstrate four predominant orientations, aligned in $0^{\circ }, 90^{\circ }, 45^{\circ }$ and $135^{\circ }$ with respect to the horizontal direction.
2. Limbal zone ($${r}>R_{\mathrm{out}}$$):
  In the region around the edge of the cornea (limbus), it is assumed that collagen fibers are highly aligned in circumferential directions (Girard et al. 2011). This is in agreement with preferential circumferential orientation of fibers reported in many other mammals (Hayes et al. 2007).
3. Transition zone ($${R}_{\mathrm{in}} <r<R_{\mathrm{out}}$$)
  In this area, the orientation of collagen fibrils between the central and limbal zones is linearly interpolated.

**Fig. 7:**
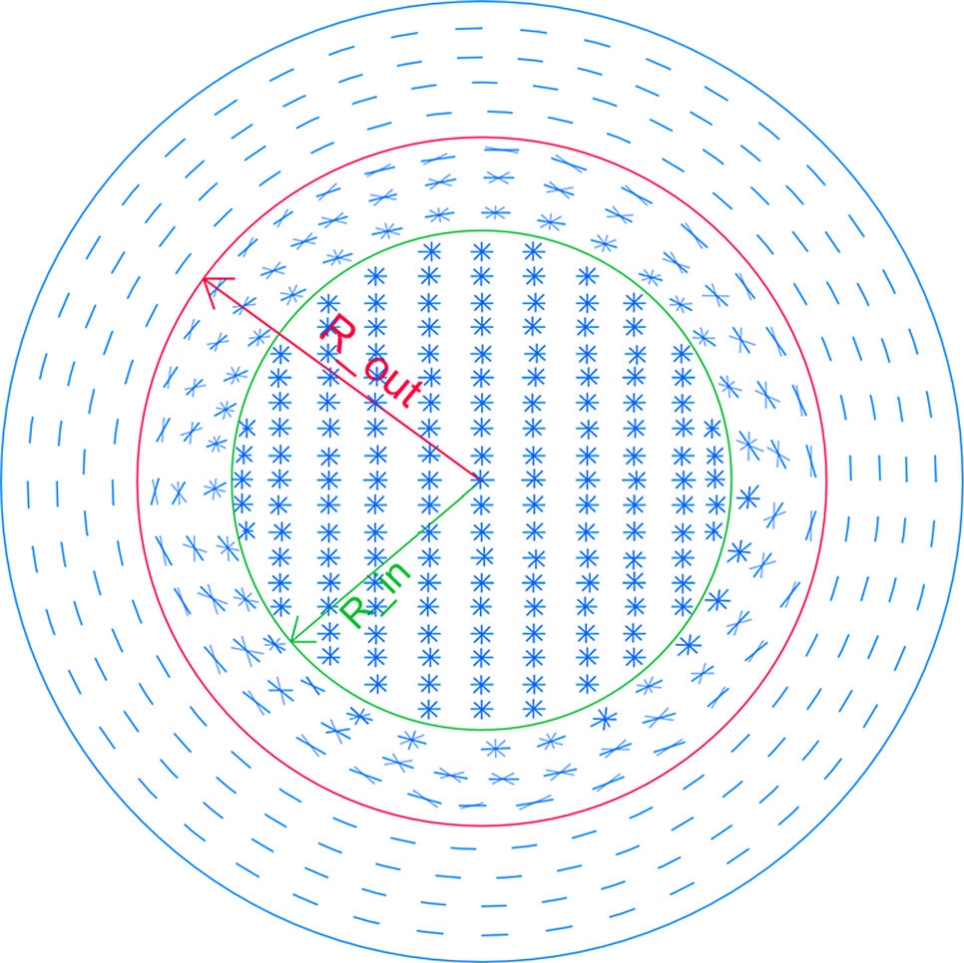
Schematic presentation of the assumed predominant directions of collagen fibrils in the rat cornea for numerical purposes. The collagen fibrils are assumed to have varying preferred orientations depending on the three regions defined on the cornea: central area, transition zone, and corneal edge

Figure 7 shows the schematic exhibition of the predominant orientations of the collagen fibrils in the three presumed regions of the rat cornea. In this cornea model, $R_\mathrm{in}$ and $R_\mathrm{out}$ are, respectively, assumed as 0.75 and 1.75 mm. These values are approximate, extrapolated from X-ray images of mouse corneas (Sheppard et al. 2010), and adjusted to the dimensions of the rat cornea.

### Collagen fibril behavior

The type I collagen forms fibers that run throughout the stroma, reinforcing and stiffening it. These fibers have well-documented nonlinear behavior, becoming stiffer with increasing strain. This behavior has been explained by the fact the molecules unwind at low strains, with relatively low resistance. At higher strains, the molecule cannot unwind further, and the backbone of the molecule is stretched, increasing resistance. Several models have been proposed for stress–strain relationship of collagen fibers [Foster et al. (2013), and references therein]. In this investigation, we do not model each fiber explicitly, but fit the overall effect of the fibers with an exponential function which stiffens with increasing strain, following Pandolfi and Holzapfel (2008).

### Constitutive model

The corneal tissue demonstrates highly nonlinear mechanical behavior. In the present work, the material model used is an anisotropic hyperelastic model adopted from Pandolfi and Holzapfel (2008) for the human cornea. The model follows the decomposition of the strain energy function into a volumetric part and isochoric components of oriented fibrils distributed in Neo-Hookean matrix:

1 $$\begin{aligned} \varPsi ( J,\mathbf{C}^\mathrm{iso} )=\varPsi ^\mathrm{iso}({\varvec{C}}^\mathrm{iso} )+U(J) \end{aligned}$$

In which $U$ is the penalty function enforcing incompressibility constraints of the cornea defined as

2 $$\begin{aligned} U(J) = \lambda \,\log ^{2}J \end{aligned}$$

Here $\lambda$ is a positive penalty parameter, and as it approaches infinity, the constraint condition is fully satisfied. A very high value of $\lambda$, however, could lead to an ill-conditioned stiffness matrix. $J$ is the Jacobian, which is the ratio of the volume in the current configuration to reference configuration $(J = \hbox {det}({\varvec{F}}), {\varvec{F}}$ is the deformation gradient).

The isochoric component is expressed as combined strain energy density of hydrated matrix $(\Psi _\mathrm{matrix}^\mathrm{iso})$ and anisotropic collagen fibrils $(\varPsi _\mathrm{fibril}^\mathrm{iso})$:

3 $$\begin{aligned}&\varPsi ^\mathrm{iso}\left( \mathbf{C}^\mathrm{iso},M_4, M_6, M_8, M_{10} \right) \nonumber \\&\quad = \varPsi _\mathrm{matrix}^\mathrm{iso}(I_{1}^\mathrm{iso})+\varPsi _\mathrm{fibril}^\mathrm{iso}(I_{1}^\mathrm{iso}, I_4^\mathrm{iso} ,I_6^\mathrm{iso} ,I_8^\mathrm{iso} ,I_{10}^\mathrm{iso}) \end{aligned}$$

Strain energy density of matrix is presented by an incompressible and isotropic Neo-Hookean model as follows:

4 $$\begin{aligned}&\varPsi _\mathrm{matrix}^\mathrm{iso} \left( {I_1^\mathrm{iso} } \right) =\frac{\mu _0 (I_1^\mathrm{iso} -3)}{2}\nonumber \\&I_{1}^\mathrm{iso}=\hbox {tr}({\varvec{C}}^\mathrm{iso})\nonumber \\&{\varvec{C}}^\mathrm{iso}=J^{-\frac{2}{3}}{\varvec{C}}\nonumber \\&{\varvec{C}}={\varvec{F}}^{T}{\varvec{F}} \end{aligned}$$

Here $\mu _{0}$ is the shear modulus analogue for the matrix, $I_{1}^\mathrm{iso}$ is the first invariant of the modified right Cauchy–Green deformation tensor ${\varvec{C}}^\mathrm{iso}$ which is the isochoric part of the second-order symmetric right Cauchy–Green tensor ***C***. Note that det$({\varvec{C}}) = J^{2}$ and hence det$({\varvec{C}}^\mathrm{iso})=1.$

Strain energy density of fibrils associated with four families of collagen fibrils is expressed as follows:

5 $$\begin{aligned}&\varPsi _\mathrm{fibril}^\mathrm{iso} (I_1^\mathrm{iso} , I_4^\mathrm{iso} ,I_6^\mathrm{iso} ,I_8^\mathrm{iso} ,I_{10}^\mathrm{iso} )\nonumber \\&\quad =\mathop \sum \limits _{i=4,6,8,10} \frac{k_1 }{2k_2 }\{\exp [k_2 ({I_{i}^\mathrm{iso}}^{*}-1)^{2}]-1\}\end{aligned}$$

6 $$\begin{aligned}&{I_{i}^\mathrm{iso}}^{*}=\kappa I_i^\mathrm{iso} +(1-3\kappa )I_i^\mathrm{iso}\end{aligned}$$

7 $$\begin{aligned}&I_{i}^\mathrm{iso}={\varvec{M}}_i \cdot {\varvec{C}}^\mathrm{iso} \cdot {\varvec{M}}_i \end{aligned}$$

Here ${\varvec{M}}_4, {\varvec{M}}_6, {\varvec{M}}_8, {\varvec{M}}_{10}$ (considering $i = 4, 6, 8, 10$) are the unit orientation vectors defined in the reference configuration that represent the mean orientation of collagen fibrils in the four directions as described earlier. $I_4^\mathrm{iso} ,I_6^\mathrm{iso} ,I_8^\mathrm{iso} ,I_{10}^\mathrm{iso}$ are pseudo-invariants that describe the square of the stretch in the directions of, respectively, ${\varvec{M}}_4, {\varvec{M}}_6, {\varvec{M}}_8, {\varvec{M}}_{10}$.

The dispersion parameter $\kappa \in [0,\frac{1}{3}]$ describes the ratio of anisotropic fibrils to isotropically distributed ones in the cornea (Gasser et al. 2006; Cortes et al. 2010).

For the rat cornea model, in the region around the cornea center, it is assumed in here that $\kappa$ = 0.066 and $\kappa$ = 0.033 around the cornea edge. In the transition region, linear interpolation of the two values is assumed. This may be written as:

8 $$\begin{aligned}&r<R_\mathrm{in} \Rightarrow \kappa = 0.066\nonumber \\&r>R_\mathrm{out} \Rightarrow \kappa = 0.033\nonumber \\&R_\mathrm{in} <r<R_\mathrm{out} \Rightarrow \kappa = 0.066 -\frac{r-R_\mathrm{in} }{R_\mathrm{out} -R_\mathrm{in} } \times 0.033 \end{aligned}$$

Also, $k_{1}$ (stress-like parameter) and $k_{2}$ (dimensionless parameter) are material parameters that define the stiffening effect of the fibrils and are determined from mechanical tests. The values of the material parameters used in the simulation are given in Table  3 [based on material parameters suggested in Pandolfi and Holzapfel (2008)] .

**Table 3:** Material constants assumed for the rat cornea model [based on data obtained in Pandolfi and Holzapfel (2008)]

$\lambda$ (kPa)	$\mu _{0}$ (kPa)	$k_{1}$ (kPa)	$k_{2}$
5500	60	20	400

It is worth noting that $${k}_2 $$ and especially $${k}_1$$ need not to be the same for all orientations, but we assume it is the case in this model.

These types of models based on several preferred collagen fiber orientations, originally suggested by Gasser et al. (2006), are computationally efficient but have some limitations. It has been shown (Cortes et al. 2010) that for somewhat dispersed orientations, the models can be inaccurate for large strains. Optimally, the distribution of fiber orientations should be integrated over a directional distribution function (Pinsky et al. 2005; Nguyen et al. 2008). In some cases, closed-form solutions exist for approximated distributions. Otherwise, the evaluation of these functions can be quite time-consuming for finite element simulations. Recently, nonlinear mappings of approximate integral (e.g., Alastrué et al. 2009) have been applied to biological tissues to make the solution of general integrals more efficient. On the other hand, second-order structure tensors (Pandolfi and Vasta 2012) have been developed to more accurately account for variation in the directional response beyond simple averaging. In the current study, the dispersion parameter is fairly small and the stress is close to biaxial and equal in plane, which should lead to acceptable error, especially in the center where the spiraling occurs. In addition, the four directions of fiber lead to a more isotropic in-plane response, further reducing the inaccuracy of the method. The model is based on limited data, and the larger source of error is likely uncertainty in the material and geometrical parameters of the rat cornea itself. Improvements will be made as more detailed data on the rat cornea is available.

The total stress developed in the cornea due to penalty function, matrix, and fibril is expressed as

9 $$\begin{aligned} {\varvec{S}}={\varvec{S}}_\mathrm{penalty} +{\varvec{S}}_\mathrm{matrix} +{\varvec{S}}_\mathrm{fibril} \end{aligned}$$

where ***S*** is the Second Piola–Kirchhoff stress for the cornea in which the constituents are derived from

10 $$\begin{aligned} {\varvec{S}}_\mathrm{penalty}&= 2 \quad \frac{\partial U}{\partial {\varvec{C}}}=2\lambda {\varvec{C}}^{-1} \log J\end{aligned}$$

11 $$\begin{aligned} {\varvec{S}}_\mathrm{matrix}&= 2 \frac{\partial \varPsi _\mathrm{matrix}^\mathrm{iso} }{\partial {\varvec{C}}}\nonumber \\&= \mu _{0} I_3^{-1/3} \left( \mathbf 1 -\frac{1}{3}I_1 {\varvec{C}}^{-1}\right) \end{aligned}$$

12 $$\begin{aligned} {\varvec{S}}_\mathrm{fibril}&= 2 \frac{\partial \varPsi _\mathrm{fibril}^\mathrm{iso} }{\partial {\varvec{C}}} =2\sum _{i=4,6,8,10} k_1 \nonumber \\&\quad \times \hbox {exp}\left[ {k_2 ({I_{i}^\mathrm{iso}}^{*}-1)^{2}} \right] \left( {I_{i}^\mathrm{iso}}^{*}-1 \right) \frac{\partial {I_{i}^\mathrm{iso}} ^{*}}{{{\partial }} {\varvec{C}}} \end{aligned}$$

where

13 $$\begin{aligned} \frac{\partial {I_{i}^\mathrm{iso}}^{*}}{{\varvec{\partial }}{\varvec{C}}}&= I_3^{-1/3} \left[ \kappa \left( {{\varvec{1}}-\frac{1}{3}{\varvec{C}}^{-1}I_1} \right) \right. \nonumber \\&\quad \left. +\left( 1-3\kappa \right) \left( {\varvec{M}}\otimes {\varvec{M}}-\frac{1}{3}{\varvec{C}}^{-1}I_i\right) \right] \end{aligned}$$

**Fig. 8:**
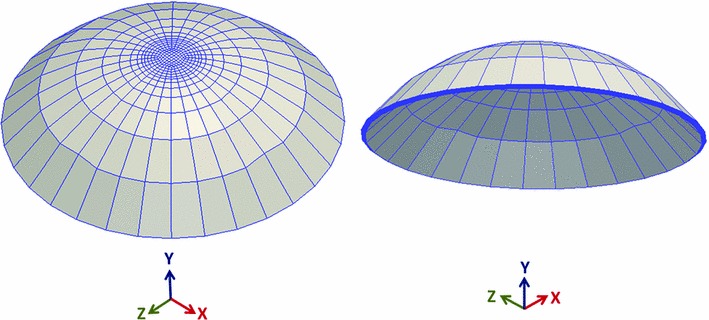
Three-dimensional representation of the 10-layered rat cornea mesh having 4480 elements using tri-linear averaged volume ${\overline{{\varvec{B}}}}$ hexahedral elements

In these equations, ***1*** is the second-order identity tensor, i.e., ${\varvec{1}}_{ij} =\delta _{ij}$, where $\delta _{ij}$ is the Kronecker delta. The “$\otimes$” symbol represents the outer product, e.g., for two vectors ***U*** and ***V***, $({\varvec{U}} \otimes {\varvec{V}})_{ij}= U_{i}V_{j}$ and for two second-order tensors ***A*** and ***B***, $({\varvec{A}} \otimes {\varvec{B}})_{ijkl }= A_{ij }B_{kl}.$

### Finite element discretization

In the analysis of nearly incompressible materials (such as the cornea), very little change in volume is observed even under large deformations. Standard 8-node hexahedra are known to exhibit volumetric locking behavior. Many solutions have been proposed to relieve the incompressibility constraints, among which the ${\overline{{\varvec{B}}}}$ method is a popular solution to relieve volumetric locking. In these elements, the volumetric part of the strain–displacement matrix, ***B***, is replaced by a reduced -order integration or averaged value (Hughes 1980). In the large deformation regime, the gradient of deformation, ***F***, is replaced by a modified deformation gradient ${\overline{{\varvec{F}}}}$ to treat incompressibility constraints (called ${\overline{{\varvec{F}}}}$ method). Here, we modify the volumetric part of the deformation gradient using an averaged integral. The integral-averaged ${\overline{{\varvec{F}}}}$ approach leads to a so-called ${\overline{{\varvec{B}}}}$ element.

The proposed averaged volume 8-node hexahedral ${\overline{{\varvec{B}}}}$ elements (with Jacobian averaged in the reference configuration) were used in meshing the structure of the rat cornea model. The elements were arranged in a radial pattern to better capture the spiral patterns near the center. The mesh was next refined throughout the thickness to assess convergence. Four different meshes each consisting of 4, 6, 8, and 10 layers of elements, respectively, having 1792, 2988, 3584, and 4480 hexahedral elements were investigated. Figure 8 shows the three-dimensional representation of the 10-layered rat cornea mesh.

## Spiral post-processing and measurements

### In-plane strain determination

We examine strains on the surface of the stroma to see how these influence the deformation of the epithelium. The epithelium does not have nearly the stiffness or strength of the stroma, and hence, the strain in the epithelium follows that of the stroma beneath. Because the epithelium has a significant viscous component to its deformation, it is difficult to know the exact stress. But the deformation likely follows that of the stroma. The epithelium is not highly anisotropic, and the strain in the epithelium is more nearly coaxial with the stress. Hence, directions of critical strain are close to those of critical stress.

The initial assumption taken is that the epithelial cells align to the direction of maximum shear strain vectors as they migrate toward the center of the cornea. Here, the determination of these vectors is briefly discussed.

The strain used in the finite element model is the Eulerian logarithmic strain defined as

14 $$\begin{aligned} {\varvec{\varepsilon }} =\log (\sqrt{{\varvec{FF}}^{T}}) \end{aligned}$$

To analyze the effects of the deformation of the stroma on the epithelium above, we restrict ourselves to the strain ${\hat{{\varvec{\varepsilon }}}}$ in the plane of the cornea surface. If ${\hat{\varepsilon }}_{1}$ and ${\hat{\varepsilon }}_{2}$ are the principal strains in the surface, then the maximum shear strain is

15 $$\begin{aligned} {\hat{\varepsilon }}_{12} =\frac{{\hat{\varepsilon }}_{1} -{\hat{\varepsilon }}_{2}}{2} \end{aligned}$$

And if ${\hat{{\varvec{n}}}}_1$ and ${\hat{{\varvec{n}}}}_{2}$ are the principal strain direction vectors belonging to, respectively, ${\hat{\varepsilon }}_1$ and ${\hat{\varepsilon }}_{2}$, the two perpendicular maximum shear strain direction vectors are defined as

16 $$\begin{aligned} {\hat{{\varvec{t}}}}=\frac{{\hat{{\varvec{n}}}}_{1} \pm {\hat{{\varvec{n}}}}_{2}}{\sqrt{2}} \end{aligned}$$

The resulting shear strain vectors point along the surface of the cornea.

As previous work on mouse cornea (Rhee et al. in review) demonstrated, directions of maximum shear strain do not always fit observed spirals well. Hence, we examine in this work a linear combination of shear and tensile strains creating critical directions, which is fully justified in Sect. 6. The critical direction may be written as a combination of the principal in-plane directions.

17 $$\begin{aligned} {\hat{{\varvec{l}}}}=\frac{{\hat{{\varvec{n}}}}_{1} \pm c\, {\hat{{\varvec{n}}}}_{2}}{||{\hat{{\varvec{n}}}}_{1} \pm c\,{\hat{{\varvec{n}}}}_{2}||} \end{aligned}$$

In this equation, $c>1$ indicates a positive effect of tension on this critical direction. The larger the value of $c$, the greater the effect of tension over shear. Including this factor in the proposed path determination algorithm leads to a curve that can be fitted with logarithmic spiral having smaller pitch angles, similar to the spirals observed on the mouse and rat corneas.

**Fig. 9:**
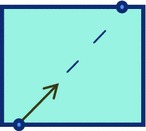
Schematic two-dimensional representation of the approach used for finding the endpoints at each element

**Table 4:** Convergence rates studied for maximum vertical displacement and maximum shear strain obtained from FE simulation

No. of layers in the mesh	No. of elements in the mesh	Maximum displacement in vertical direction (mm)	Maximum shear strain
4	1,792	0.23603	0.10705
6	2,688	0.24124	0.10746
8	3,584	0.24126	0.10772
10	4,480	0.24127	0.10788

The maximum vertical displacement converges more rapidly

**Fig. 10:**
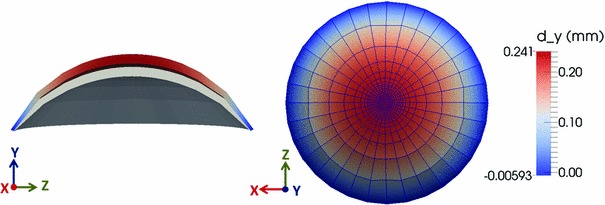
Vertical displacement mapping of the rat cornea subjected to IOP is shown in cross section on the *left* and on the *top surface* on the *right*. In the *left picture*, the *gray surface* is the undeformed shape of the cornea and vertical displacement *colored* by magnitude is shown in *top*

### Pathline determination

To quantify the spiral patterns on the cornea surface created by the vector field critical strains, an algorithm was devised to track the pathlines of in-plane critical strain directions. In this procedure, starting from an arbitrary point on any element on the topmost layer, critical strain vectors are traced to the adjacent element.

Within each element, the spiral pattern is approximated using a straight line by averaging the strain, requiring only the determination of two endpoints in each element in space (refer to Fig. 9). The endpoints are found by matching the equation of the critical strain direction in the element to the equations of the element edges [similar to the approach used in Foster et al. (2007), Foster and Mohammad Nejad (2011)]. The endpoints are next connected together toward the center of the cornea, forming a curve that resembles spiral patterns. As the mesh refinement is performed, more accurate spiral-like curves are obtained. Since there are two critical strain directions, we select the one that matches the spiral we wish to fit, either clockwise inward or counter clockwise inward.

**Table 5:** For each rat, the pitch angle of the logarithmic spiral observed on the cornea is measured

Cornea label	Pitch angle ($^{\circ }$)	$c$
C1	-42.7	1.00
C2	32.5	1.45
C3-1	23.4	2.00
C3-2	24.0	2.00

The constant ($c$) is selected to fit the simulation curve to the observed spiral

## Results and measurements

To verify convergence, two quantities are examined, the maximum displacement in the vertical direction and the maximum logarithmic shear strain (see Table 4). As observed in the table, the maximum displacement converges rapidly toward a single value. The maximum shear strain also converges, but with a slower rate as expected.

**Fig. 11:**
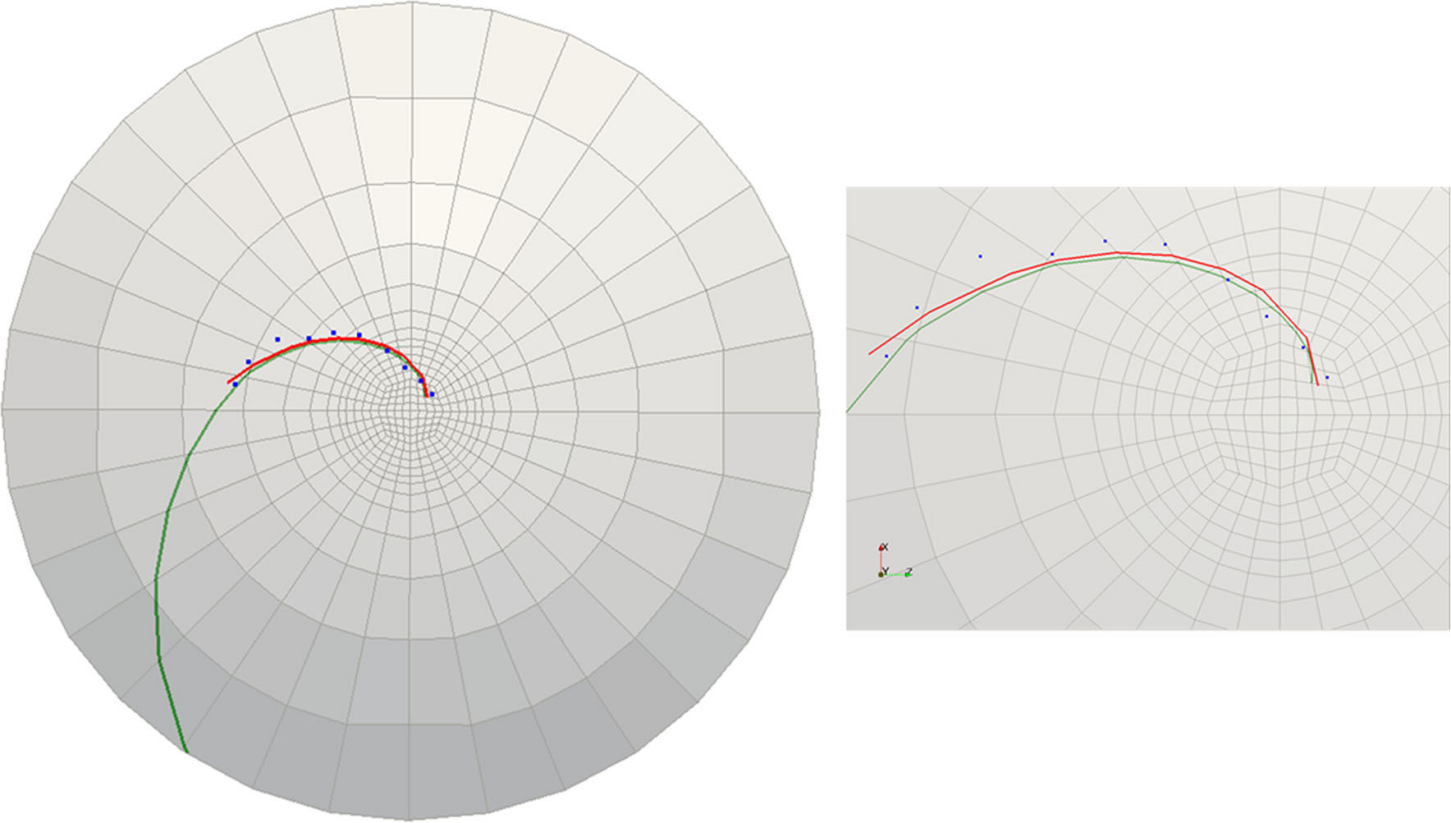
Cornea C1: landmark points overlaid onto the cell boundaries are shown in *blue*, the logarithmic spiral with $-42.7^{\circ }$ pitch angle fitted to the landmarks is shown in *red*, and *green curve* is obtained from FE simulations ($c$ = 1). The figure on the *right* demonstrates the *zoomed-in* view of the central part of the rat cornea on the *left*

**Fig. 12:**
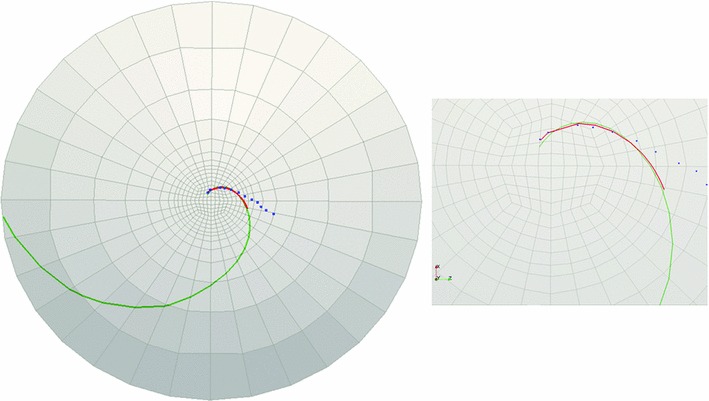
Cornea C2: landmark points overlaid onto the cell boundaries are shown in *blue*, the logarithmic spiral with $32.5^{\circ }$ pitch angle fitted to the landmarks is shown in *red*, and *green curve* is obtained from FE simulations ($c$ = 1.45). The figure on the *right* demonstrates the *zoomed-in view* of the central part of the rat cornea on the *left*

**Fig. 13:**
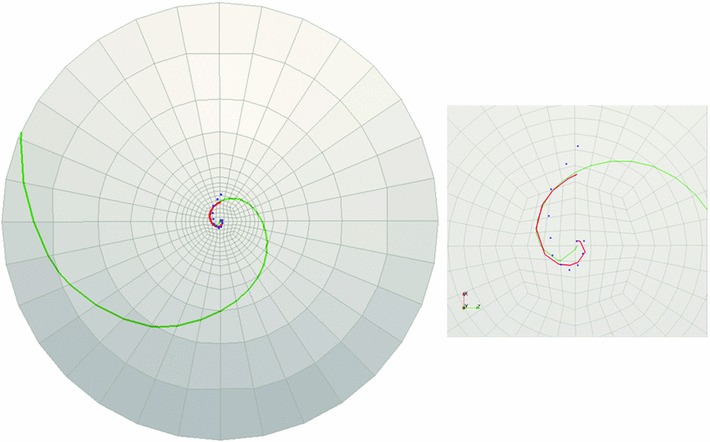
Cornea C3-1: landmark points overlaid onto the cell boundaries are shown in *blue*, the logarithmic spiral with $23.4^{\circ }$ pitch angle fitted to the landmarks is shown in *red*, and *green curve* is obtained from FE simulations ($c$ = 2). The figure on the *right* demonstrates the *zoomed-in view* of the central part of the rat cornea on the *left*

**Fig. 14:**
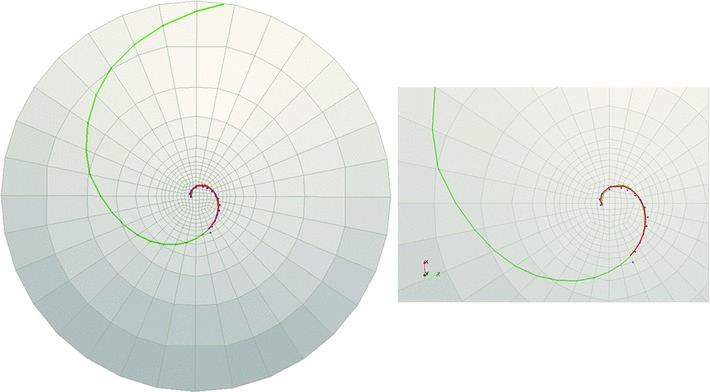
Cornea C3-2: landmark points overlaid onto the cell boundaries are shown in *blue*, the logarithmic spiral with $24^{\circ }$ pitch angle fitted to the landmarks is shown in *red*, and *green curve* is obtained from FE simulations ($c$ = 2). The figure on the *right* demonstrates the *zoomed-in* view of the central part of the rat cornea on the *left*

**Fig. 15:**
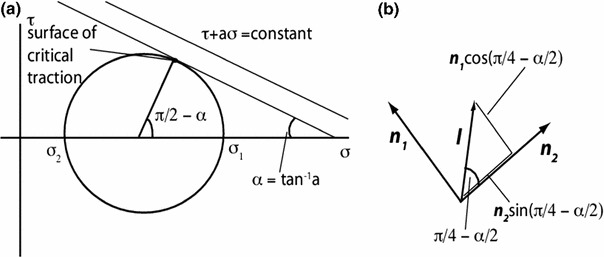
**a** Mohr’s circle showing the critical surface traction, located at $\frac{\pi }{2}-\alpha$ from the major principal strain on Mohr’s circle, or half that in physical space. **b** Critical direction in physical space as a function of the principal directions

Figure 10 illustrates the deformed shape and vertical displacement mapping of the rat cornea subjected to IOP on the 10-layered mesh with 4480 elements obtained from FE simulation.

### Comparison of observed spirals and simulation results

As explained earlier, four adult rat corneas (two from the same rat) were sectioned and imaged with confocal microscopy. Landmark points were overlaid onto the cell boundaries that resembled spiral patterns. These points were fitted with logarithmic spirals and the spiral pitch angles were measured. For each rat cornea, the logarithmic spiral with median pitch angle was selected to be compared to the finite element simulation results.

The pathline determination algorithm was performed to trace the pathlines of in-plane critical strain directions obtained from finite element simulation. For each rat cornea, these directions were tracked starting from a point close to the beginning point of the logarithmic spiral fitted to the observed patch edges. However, the pathlines of maximum shear strains did not fit the observed logarithmic spirals well in all of the sampling corneas. In some cases, in order to find the best fit to the observed logarithmic spirals, the pathline tracking algorithm was modified by considering a combination of the effect of shear and tensile strains. The details will be discussed in Sect. 6. Table 5 demonstrates the constants used to fit the simulation curves to the logarithmic spiral observed at each cornea and the measured spiral pitch angles. Figures 11, 12, 13, and 14 demonstrate the landmark points overlaid onto the cell boundaries, the logarithmic spirals fitted to the landmarks and spiral-like curves obtained from numerical simulation (on the 10-layered mesh with 4480 elements) on rat corneas C1, C2, C3-1, and C3-2, respectively.

## Discussion

In young rats, the cornea likely inflates as the IOP increases. Though we have not explicitly modeled it, the cornea may also grow during this phase. The resulting deformations are likely similar. In either case, the epithelial cells must move and grow to maintain coverage of the cornea. The cells do not appear to fracture, and hence must slide and stretch past each other as they move to cover the surface. The shear strains in the Bowman’s membrane facilitate this kind of sliding. As the cells slide past, like cells tend to have stronger adhesion [Foty and Steinberg (2005), and references therein], and tend to stick as cells continue to slide past each other.

However, pathlines of maximum shear strain do not fit observed spirals well in most cases. We hypothesize that normal strain influences the ability of cells to slide with respect to each. Such behavior is observed in soils and other materials, though typically the normal stress is compressive in these cases. In the cornea, larger tension on cell boundaries, tending to pull the cells apart, may facilitate sliding.

While there is discussion of both tensile and shear deformation in the cell in tissue mechanics literature, there is little discussion of how normal components might influence shear deformation. The deformation of epithelial cells under tension in Wiebe and Brodland (2005) suggests, however, the tension-facilitated shear may be at work in cell deformation. Before fracturing in tension, the cells exhibit noticeable distortion. In pure shear sliding, which is sometimes seen in metals, the slip planes would be at $45^{\circ }$ from the maximum tensile stress. In the figures presented, the angle appears lower, approximately $21^{\circ }$ from the plane of maximum principal stress. This suggests that a mix of shear and tension leads to the critical slip direction in these cells.

**Fig. 16:**
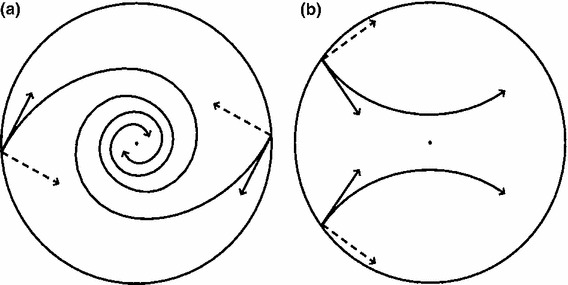
**a** If the majority of cells in all parts of the cornea tend to slip in one dominant direction, clockwise or counterclockwise, the cells will spiral. **b** If cells in one section start to slide in a different direction that other cells, different patterns, such as horseshoes, can emerge

Unlike the stroma, the epithelium is more or less isotropic, at least in the plane of the cornea surface. Hence, stresses and strains are coaxial. If we consider a linear effect of tension on shear motion, then motion most likely occurs, overall, on the plane of maximum

18 $$\begin{aligned} \left| {\tau } \right| +a\sigma \end{aligned}$$

where $\tau$ is the shear stress, and $\sigma$ is the normal stress. Using Mohr’s circle (Fig. 15a), we can see that the critical direction is $\pm \left( {\frac{\pi }{4}-\frac{\alpha }{2}} \right)$ from the direction of the smaller principal stress, where tan $\upalpha =a$. Therefore, as shown in Fig. 15b, the critical direction

19 $$\begin{aligned} l&= \sin \left( {\frac{\pi }{4}-\frac{\alpha }{2}} \right) n_{1} \pm \cos \left( {\frac{\pi }{4}-\frac{\alpha }{2}} \right) n_{2}\nonumber \\ l&= \frac{n_{1} \pm \cot \left( {\frac{\pi }{4}-\frac{\alpha }{2}} \right) n_{2} }{n_{1} \pm \cot \left( {\frac{\pi }{4}-\frac{\alpha }{2}} \right) n_{2} } \end{aligned}$$

Hence

20 $$\begin{aligned} c=\cot \left( {\frac{\pi }{4}-\frac{\alpha }{2}} \right) =\tan \left( {\frac{\pi }{4}+\frac{\alpha }{2}} \right) =a+\sqrt{1+a^{2}} \end{aligned}$$

The last identity comes from some trigonometric manipulation. Since we are more concerned with directions in this study, we will focus on manipulating $c$ rather than $a$. However, a linear relationship between the effect of normal and shear stress leads to a critical direction that is a linear combination of the two principal directions.

This evidence for this type of model is, of course, preliminary. The cells in Wiebe and Brodland (2005) are not corneal epithelial cells. The relationship between normal and shear stress may be nonlinear, though a linear model may be a good first approximation. Cell boundaries have fairly random orientations, and hence, the normal and shear stress vary across a line of moving cells. In this work, we are attempting to understand the tissue-level slip patterns of the cells, but multiscale efforts may reveal the variation that different initial configurations create with regard to the patterns observed. In this regard, the cell motion over time may have a significant effect. On a planar surface under uniaxial tension, the $45^{\circ }$ slip lines would only become apparent after large deformations. Similarly, motion of cells in the cornea creates an evolution of patterns that has not been fully investigated. Although the sample size here is quite small, the spirals seem show a tendency to tighten over time. This type of behavior seems to make sense as the cells continue to arrange themselves over time. Further investigation is necessary to confirm this observation. Cell growth, division, and death along with the viscous behavior of the cell matrix may also influence the deformation patterns.

It is worth noting that, with or without a tensile component, there are two critical slip directions. If the majority of cells throughout the cornea tend to slip in one dominant direction, clockwise or counterclockwise, the cells will spiral (see Fig. 16a). Presumably, there is no preferred direction and initial perturbations determine the orientation of the spirals. If cells in one section start to slide in a different direction, and cells in a different part of the cornea start to slide in the other, different patterns, such as horseshoes, can emerge (Fig. 16a). Such patterns have been observed in corneas.

## Summary and conclusion

During the development of an organ, stem cells assort themselves into patterns as a result of cell division, cell movement, and cell death. These processes operate in a typical reproducible and conserved manner. If the organ is comprised of two or more genetically distinct groups of cells, the distribution of cells can be visually observed. In some mammalian corneas such as mice and rats, the allocation of epithelial cells is followed by a special assortment of cells under both normal and diseased conditions. The edges of patches of epithelial cells having similar lineage often form distinctive spiral patterns that can be visualized. Studying the formation of such patterns is important as it can reveal greater understanding of corneal function and possible disorders. This paper proposes a framework for explaining the development of epithelial cells into spiral patterns due to the effect of stresses and strains on the cornea.

Confocal images of four adult rat corneas (two from the same rat) demonstrating spiral patterns were provided. Landmark points were overlaid onto the patch edges. Logarithmic spirals were fitted to the points, and the spiral pitch angles were measured. For each rat cornea, the logarithmic spiral with median pitch angle was compared to the finite element simulation results.

It was initially assumed in this work that shear strains in the tissue facilitate the sliding of epithelial cells past each other and cause special assortment of cells into spiral patterns. An algorithm was devised to track the pathlines of critical strain directions obtained from finite element simulation. The resulting curve matched the observed logarithmic spirals well for sampling rat cornea C1. The algorithm was modified considering a combination of the effect of shear and tensile strains for other rat corneas samples. In the corneas C2, C3-1, and C3-2, a proper combination of shear and tension was selected to fit well to the observed spirals. Interestingly, the selected combination was the same for cornea C3-1 and C3-2 as they both belong to one rat. This suggests that in the same animal, corneal epithelial cells form similar patterns. This has been observed previously although in some examples, the spiral is clockwise in one cornea and counter clockwise in the other.

This work presented a framework for studying the development of spiral patterns in the rat epithelium. Using the finite element method as a basis, this framework included a detailed structural and constitutive representation of the rat cornea. A continuum finite deformation model considering material and geometric nonlinearity of collagen fibrils and incompressibility of hydrated matrix was developed. The proposed model included a ${\overline{{\varvec{B}}}}$ method to treat incompressibility constraints of the cornea in the large deformation regime. The model also involved the effects of preferentially oriented and dispersed collagen fibrils throughout the rat stroma. Based on the simulation results, a mesh of ten layers achieved converged values of strain, though the displacement convergence was much faster. Good agreement between the simulation curves and logarithmic spirals fitted to confocal images of the rat cornea were obtained. This supported the proposed assumption that shear and tensile strains facilitate sliding of epithelial cells.

There are some factors affecting the motion of epithelial cells that were not considered in our numerical model. Variations in the spiral development due to the cell growth and division in the organ were not included in our model. The spiral may also evolve with age. Dynamic motion of the cornea caused by the changing intraocular pressures was lacking in our model. The material parameters used in this rat cornea model were based on limited data, and improved understanding of the structure of the rat cornea could modify the results.

Future directions include changing the shape of the surface of the stroma as an input condition to model the rearrangement of epithelial cells over time. Cellular finite element models of tissues exist that account for the adhesion of the membranes, the viscosity of the cellular fluid, and even cell growth and division. More detailed models of the shear and tensile behavior of cell-to-cell adhesion would need to be developed, as well as the attachment to the basement membrane.

## Appendix: Tangent modulus

The fourth-order tangent modulus tensor ***D*** in the cornea is stated as:

21 $$\begin{aligned}&{\varvec{D}}={\varvec{D}}_\mathrm{penalty} +{\varvec{D}}_\mathrm{matrix} +{\varvec{D}}_\mathrm{fibril}\end{aligned}$$

22 $$\begin{aligned}&{\varvec{D}}_\mathrm{penalty} =4 \frac{\partial ^{2}{\varvec{U}}_\mathrm{penalty} }{\partial {\varvec{C}}^{2}}=2\mu _{0} (-{\varvec{L}}\log J + {\varvec{C}}^{-1} \otimes {\varvec{C}}^{-1})\nonumber \\ \end{aligned}$$

in which ${\varvec{L}}=\frac{\partial {\varvec{C}}^{-1}}{\partial {\varvec{C}}}$ is a fourth-order tensor

23 $$\begin{aligned} {\varvec{D}}_\mathrm{matrix}&= 4\frac{\partial ^{2}\varPsi _\mathrm{matrix}^\mathrm{iso} }{\partial {\varvec{C}}^{2}}=\frac{2\mu _{0} I_3^{-1/3} }{3}\nonumber \\&\quad \times \left( \frac{I_1 }{2} {\varvec{L}} + \frac{1}{3}I_1 {\varvec{C}}^{-1} \otimes {\varvec{C}}^{-1} - {\varvec{1}}{\varvec{C}}^{-1}-{\varvec{C}}^{-1}\otimes {\varvec{1}}\right) \end{aligned}$$

24 $$\begin{aligned} {\varvec{D}}_\mathrm{fibril}&= 4\frac{\partial ^{2}\varPsi _\mathrm{fibril}^\mathrm{iso} }{\partial {\varvec{C}}^{2}}\end{aligned}$$

25 $$\begin{aligned} {\varvec{D}}_\mathrm{fibril}&= 4k_1 \sum \nolimits _{i=4,6,8,10} \hbox {exp}(k_2 ({I_{i}^\mathrm{iso}}^{*}-1)^{2})\nonumber \\&\quad \times \left\{ [1+2k_2 ({I_{i}^\mathrm{iso}}^{*}-1)^{2}]\frac{\partial {I_{i}^\mathrm{iso}}^{*}}{\partial {\varvec{C}}}\otimes \frac{\partial {I_{i}^\mathrm{iso}}^{*}}{\partial {\varvec{C}}}\right. \nonumber \\&\quad \left. + (I_{i}^\mathrm{iso}- 1)\frac{\partial ^{2}{I_{i}^\mathrm{iso}}^{*}}{\partial {\varvec{C}}^{2}}\right\} \end{aligned}$$

where

26 $$\begin{aligned} \frac{\partial ^{2}I_{{i}^\mathrm{iso}}^{*}}{\partial {\varvec{C}}^{2}}&= \kappa \frac{\partial ^{2}I_{1}^\mathrm{iso}}{\partial {\varvec{C}}^{2}}+(1-3\kappa ) \frac{\partial ^{2}I_{i}^\mathrm{iso}}{\partial {\varvec{C}}^{2}}\end{aligned}$$

27 $$\begin{aligned} \frac{\partial ^{2}I_1^\mathrm{iso}}{\partial {\varvec{C}}^{2}}&= -\,\frac{1}{3}I_3^{-\frac{1}{3}} \left\{ {{\varvec{1}}} \otimes {\varvec{C}}^{-1}-\frac{1}{3}I_1{\varvec{C}}^{-1}\otimes {\varvec{C}}^{-1}\right. \nonumber \\&\quad \left. +\,{\varvec{C}}^{-1}\otimes {{\varvec{1}}}- {\varvec{L}}\otimes I_1\right\} \end{aligned}$$

28 $$\begin{aligned} \frac{\partial ^{2}I_1^\mathrm{iso}}{\partial {\varvec{C}}^{2}}&= -\,\frac{1}{3}I_3^{-\frac{1}{3}}\left\{ {\varvec{M}}_{{{\varvec{i}}}} \otimes {\varvec{M}}_i \otimes {\varvec{C}}^{-1}-\frac{1}{3}I_i{\varvec{C}}^{-1}\right. \nonumber \\&\quad \left. +\,{\varvec{C}}^{-1}\otimes {\varvec{M}}_{{\varvec{i}}} \otimes {\varvec{M}}_i -\frac{1}{2}I_i {\varvec{L}}\right\} \end{aligned}$$
